# Retinal Deposits of TDP‐43 and Amyloid Beta and Associated Neurodegenerative Diseases are Accurately Classified using Measured Interactions with Polarized Light in Machine Learning Algorithms

**DOI:** 10.1002/alz70861_108465

**Published:** 2025-12-23

**Authors:** Melanie CW Campbell, Lyndsy Acheson, Erik L Mason, Tanya Hareesha Shetty, Laura Emptage, Rachel Redekop, Monika Kitor, Ian R MacKenzie, Naomi C Futhey, Veronica Hirsch‐Reinshagen, Ging‐Yuek Robin Hsiung

**Affiliations:** ^1^ University of Waterloo, Waterloo, ON Canada; ^2^ University of British Columbia, Vancouver, BC Canada

## Abstract

**Background:**

We have shown that interactions with polarized light differed significantly between retinal amyloid beta deposits, associated with Alzheimer’s disease, and deposits of TDP‐43 found in the neurodegenerative diseases Frontotemporal Lobular Dementia (FTLD) and Amyotrophic Lateral Sclerosis (ALS). Our non‐invasive retinal imaging could be the first differential diagnostic of these neurodegenerative diseases. Here, the deposits’ polarized light interactions are used in machine learning to classify the deposits.

**Method:**

Post‐mortem eyes and brains were obtained from 2 individuals with ALS, 1 of whom also had FTLD, and 4 individuals with FTLD, including 1 with Type C. Brain TDP‐43 was present in the FTLD cases and some had age‐related tau. Flat‐mounted retinas were imaged using a polarimeter and then in thioflavin fluorescence. 270 presumed amyloid beta deposits in 10 individuals who had brain amyloid beta and tau and a moderate to high likelihood of AD, and 138 presumed TDP‐43 deposits in those with FTLD and/or ALS were imaged. In 1 individual with concurrent low values of brain amyloid and 1 with FTLD‐Type C, only thioflavin negative deposits were classed as potential TDP‐43 deposits. Interactions of polarized light with deposits were analyzed. Random forest (RF), an ensemble learning method and convolutional neural networks (CNN), were then used to differentiate amyloid beta from TDP‐43 deposits.

**Result:**

The deposit means and/or standard deviations of nine different polarized light interactions were significantly different between the presumed TDP‐43 retinal deposits, found in ALS and FTLD, and amyloid deposits found in AD. With borderline SMOTE augmentation of the data, we achieved a classification accuracy of 86.5± 0.6% using random forest, utilizing 6 of these interactions. CNN achieved a classification accuracy of >96% accuracy using images of the distributions of 3 polarimetric properties.

**Conclusion:**

Machine learning, using the averages and standard deviations of polarized light properties in RF or images showing the distribution of these properties across the deposits (CNN) can differentiate retinal deposits associated with Alzheimer’s disease from those associated with ALS and FTLD, with a relatively high accuracy. This first differential diagnostic of Alzheimer's disease from TDP‐43 related diseases, is early, non‐invasive and inexpensive and would reach underserved populations.

